# Novel *Mycobacterium tuberculosis* Complex Isolate from a Wild Chimpanzee

**DOI:** 10.3201/eid1906.121012

**Published:** 2013-06

**Authors:** Mireia Coscolla, Astrid Lewin, Sonja Metzger, Kerstin Maetz-Rennsing, Sébastien Calvignac-Spencer, Andreas Nitsche, Pjotr Wojtek Dabrowski, Aleksandar Radonic, Stefan Niemann, Julian Parkhill, Emmanuel Couacy-Hymann, Julia Feldman, Iñaki Comas, Christophe Boesch, Sebastien Gagneux, Fabian H. Leendertz

**Affiliations:** Swiss Tropical and Public Health Institute, Basel, Switzerland (M. Coscolla, J. Feldman, S. Gagneux);; University of Basel, Basel (M. Coscolla, J. Feldman, S. Gagneux);; Centro Superior de Investigación en Salud Pública, Valencia, Spain (M. Coscolla, I. Comas);; Robert Koch-Institut, Berlin, Germany (A. Lewin, S. Metzger, S. Calvignac-Spencer, A. Nitsche, P. Wojtek Dabrowski, A. Radonic, F.H. Leendertz);; Max-Planck-Institute for Evolutionary Anthropology, Leipzig, Germany (S. Metzger, C. Boesch);; German Primate Center, Goettingen, Germany (K. Maetz-Rennsing);; Research Centre Borstel, Borstel, Germany (S. Niemann);; Wellcome Trust Sanger Institute, Cambridge, UK (J. Parkhill);; Laboratoire Nationale de la Pathologie Animale, Bingerville, Côte d’Ivoire (E. Couacy-Hymann);; CIBER in Epidemiology and Public Health, Barcelona, Spain (I. Comas)

**Keywords:** tuberculosis and other mycobacteria, bacteria, Mycobacterium tuberculosis, MTBC, wild chimpanzee, zoonoses, nonhuman primate, infection, lineage, M. tuberculosis, tuberculosis, TB, Africa, mammals

## Abstract

Tuberculosis (TB) is caused by gram-positive bacteria known as the *Mycobacterium tuberculosis* complex (MTBC). MTBC include several human-associated lineages and several variants adapted to domestic and, more rarely, wild animal species. We report an *M. tuberculosis* strain isolated from a wild chimpanzee in Côte d’Ivoire that was shown by comparative genomic and phylogenomic analyses to belong to a new lineage of MTBC, closer to the human-associated lineage 6 (also known as *M. africanum* West Africa 2) than to the other classical animal-associated MTBC strains. These results show that the general view of the genetic diversity of MTBC is limited and support the possibility that other MTBC variants exist, particularly in wild mammals in Africa. Exploring this diversity is crucial to the understanding of the biology and evolutionary history of this widespread infectious disease.

Tuberculosis (TB) is caused by closely related acid-fast bacteria known as the *Mycobacterium tuberculosis* complex (MTBC) (*1*). MTBC includes the typical human-associated pathogens *M. tuberculosis* and *M. africanum* (*2*); *M. canettii* and other so-called “smooth TB bacilli” (*3*), the actual host range of which remains unknown; and several lineages adapted to different mammal species that include *M. bovis*, *M. microti*, *M. caprae*, *M. orygis*, and *M. pinnipedii* (*4*–*6*). Because of the wider host range of animal-associated MTBC, the common view until a decade ago was that human TB strains had evolved from *M. bovis*, the typical agent of bovine TB. Recent comparative genomic analyses have challenged this view by showing that animal MTBC strains nest within the genetically more diverse human MTBC strains (*4*,*7*–*9*). These results not only contradict the hypothesis of an animal origin for human MTBC but also promote an alternative scenario for a human origin of animal MTBC (*10*). However, little is known about MTBC diversity in domestic animals, and even less about MTBC diversity in wildlife, including our phylogenetically closest relatives, the great apes. Of note, novel members of MTBC affecting wild mammals in Africa have recently been discovered (*11*,*12*), a finding that suggests animal MTBC is more diverse than previously thought.

We report microbiologically confirmed MTBC infection in a wild chimpanzee. We show that this infection was caused by a divergent MTBC strain that does belong to the clade that includes *M. bovis* and all other animal-associated members of MTBC but is more closely related to human-associated lineage 6 (also known as *M. africanum* West Africa type 2 [WA2]). This finding highlights critical gaps in knowledge of MTBC diversity and indicates that African wildlife, and more particularly nonhuman primates, are potential hosts of novel MTBC variants.

## Materials and Methods

### Investigation of Wild Chimpanzee Death

In the course of a long-term study comprising behavioral observations and disease investigations of wild chimpanzees habituated to humans in Taï National Park, Côte d’Ivoire, necropsies are performed routinely on any chimpanzee or other mammal found dead. Detailed analyses are performed to identify the causes of death of every animal (*13*).

On August 5, 2009, an adult female chimpanzee of one of the study communities was found dead; lesions on the throat and alarm calls by other members of the community under observation at the time indicated that the animal had been killed by a leopard. The chimpanzee was one of the oldest females of the group (estimated age 52 years), and her body condition had deteriorated over the years. Necropsy was performed and tissue samples were frozen and fixed in formalin. Frozen organ material was submerged in 70% ethanol, rinsed twice in 0.85% NaCl, shredded with a scalpel, and streaked onto Löwenstein-Jensen PACT agar (Oxoid, Cambridge, UK). Bacteria were then cultivated on Middlebrook 7H11 agar supplemented with OADC or in Middlebrook 7H9 broth supplemented with OADC (Becton Dickinson, Franklin Lakes, NJ, USA) without shaking at 37°C.

### Investigation of MTBC in Other Chimpanzees

To investigate the possible presence of MTBC strains in other chimpanzees, samples were collected from 28 chimpanzees, many from the same community, that died in the same area within the previous 10 years. DNA was extracted from 115 tissue samples (lung, spleen, liver, lymph nodes, and small intestines) from these 28 chimpanzees by using the QIAGEN DNeasy Blood and Tissue Kit (QIAGEN, Hilden, Germany). All tissues were tested in duplicate by using the primers MTC_IAC Fw and MTC_IAC Rv and MTC Probe as described (*14*). We performed the PCR-RFLP of *gyr*B using the primers MTUB-f (5′-TCGGACGCGTATGCGATATC-3′) and MTUB-r (5′-ACATACAGTTCGGACTTGCG-3′) and an annealing temperature of 65°C for the PCR. We used the DreamTaq DNA Polymerase Kit and Fermentas restriction enzymes (Thermo Scientific, Waltham, MA, USA).

### Genome Sequencing of MTBC Isolates

Mycobacterial DNA was isolated by using the CTAB method as described (*15*). The DNA was used to generate libraries for 454 and Illumina sequencing (Illumina, Inc., San Diego, CA, USA). For both libraries, the DNA was sheared to a size of 400–500 bp by using a Covaris S2 (Covaris, Inc., Woburn, MA, USA). The 454 library was generated by using the Rapid Library Kit and sequenced with Titanium chemistry on a 454 FLX instrument (Roche, Penzberg, Germany). The paired-end library for Illumina sequencing was generated by using the TruSeq DNA Sample Preparation Kit (Illumina). Cluster generation was done by using TruSeq PE Cluster Kit version 2.5 (Illumina) on a c-bot. Sequencing was performed on a HiScanSQ instrument and TruSeq SBS Kit–HS chemistry (Illumina) to generate 2 × 100 bases long paired-end reads. 

Mycobacterial strains (as defined in [*7*,*16*,*17*]) were cultured from single colonies. Genomic DNA was extracted by using a standard kit (QIAGEN) and sequenced with an Illumina Genome Analyzer. Sequencing libraries were constructed by using standard kits from Illumina, according to the manufacturer’s instructions. Libraries for each strain were loaded into a single lane of a flow cell. SYBR green assays were used to test flow cells for optimal cluster density.

### Single-nucleotide Polymorphism Calling and Genome Assembly

#### Illumina Sequencing Reads

We used BWA (*18*) to map Illumina reads from the 10 genome sequences published in this study (www.ebi.ac.uk/ena/data/view/ERP001571) and 24 genomes published previously (*18*) or available in public databases (Technical Appendix Table 1) against the MTBC reference genome. The reference genome used was an inferred common ancestor of all MTBC lineages (*19*). BWA outputs were analyzed with SAMtools (*20*). We applied heuristic filters to remove problematic positions and set Phred-scaled probability at 20. SNP lists for individual strains were combined in a single, nonredundant dataset, and the corresponding base call was recovered for each strain. After excluding single-nucleotide polymorphisms (SNPs) in genes annotated as PE/PPE, integrase, transposase, or phage and SNPs that showed an ambiguous base call, we kept 12,920 high-confidence variable positions for downstream analysis. Lineage 6 strains showed an average sequencing depth of between 80 and 204-fold whereas the chimpanzee strain was sequenced at 4428-fold coverage.

#### 454 Sequencing Reads

A combined mapping and de novo assembly was performed on the 454 reads obtained during the initial sequencing. Mapping of reads to the genome of *M. tuberculosis* strain CCDC5180 resulted in reference coverage of 98.39% and a total of 90 contigs. Mapping to the genome of strain H37Rv resulted in reference coverage of 98.3% and 87 contigs. Newbler 2.5 (Roche) and MIRA 3.0.0 (*21*) were used for de novo assembly. The parameters (minimum overlap identity and length, seed length, step and count, and alignment difference and identity scores for newbler; minimum overlap identity and length; and clip length and stringency for MIRA) were automatically optimized for contig length by using a genetic algorithm with the default parameters used as starting points, a population size of 10, and 10 generations. The best set of parameters resulted in 2,538 contigs with a maximum length of 14,183 bp and a mean length of 1,738 bp. Reassembly of the contigs obtained from both mappings and from the de novo assemblies was performed by using Geneious 5.0 (Biomatters Ltd., Auckland, New Zealand) and yielded 33 contigs with a maximum length of 435,720 bp, a mean length of 130,500 bp, and a total length of 4,306,842 bp, compared with the ≈4.4 Mbp of the reference strains. The raw reads were mapped against these contigs to eliminate assembly errors.

A total of 33 contigs resulting from assembling 454 reads were aligned respective to the MTBC reconstructed ancestor genome using MAUVE (*22*). SNP lists obtained from Illumina sequencing were verified with the 454 contig sequences.

### Phylogenetic Analysis

**Figure 1:**
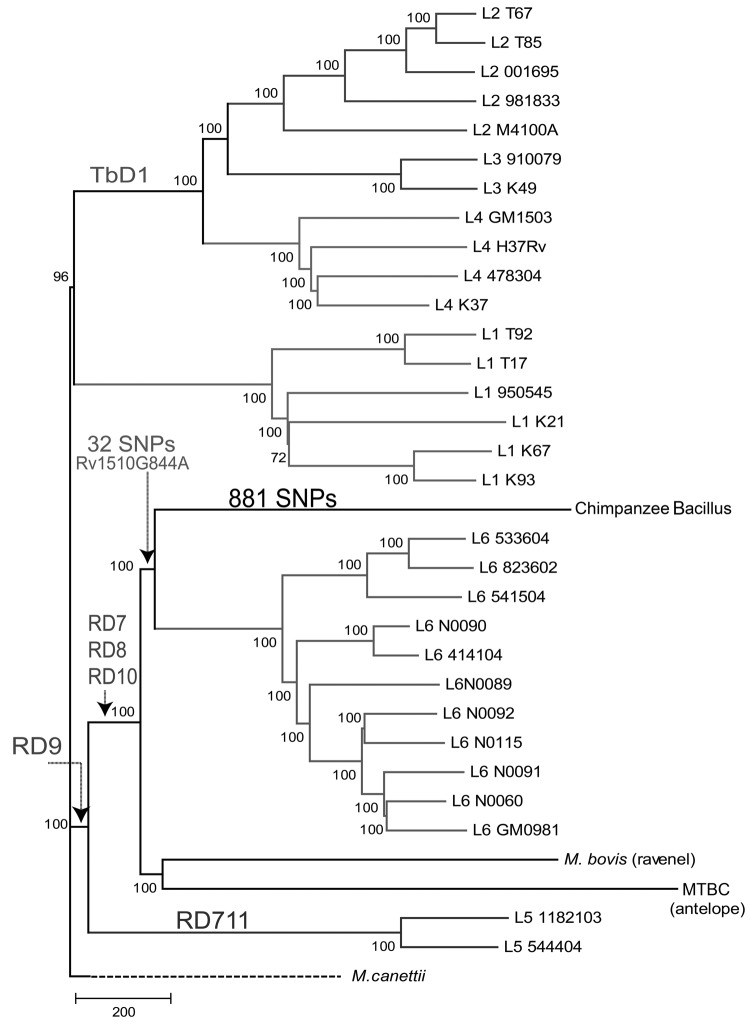
Neighbor-joining phylogenic tree constructed on the basis of 13,480 variable common nucleotide positions across 36 human and animal *Mycobacterium tuberculosis* complex (MTBC) genome sequences, including 21 previously published genomes (*18*) and the MTBC strain isolated from an adult female chimpanzee that was found dead in Taï National Park, Côte d’Ivoire, on August 5, 2009 (Chimpanzee Bacillus). The tree is rooted with *M. canettii*, the closest known outgroup. Node support after 1,000 bootstrap replications is indicated. Genomic deletions identified in (*7*) are indicated. The number of single-nucleotide polymorphisms (SNPs) exclusive of the chimpanzee strain is indicated in the respective branch, and the number of SNPs shared with the most closely related group of strains is indicated in the common branch. Scale bar indicates number of SNPs. This tree is congruent with the maximum-likelihood phylogeny shown in Technical Appendix Figure 2.

Phylogenetic analysis was performed on the basis of 13,480 high-confidence variable positions, specifying *M. canettii* as the outgroup (Figure 1). Both coding and noncoding SNPs were included. The SNPs were used to infer the phylogenetic relationships between strains by using neighbor-joining (Figure 1), maximum-likelihood (ML; Technical Appendix Figure 2), and Bayesian (Technical Appendix Figure 2) methods. Because of the low number of homoplasies expected (*18*), a neighbor-joining tree was obtained by using MEGA5 (*23*), with observed number of substitutions as a measure of genetic distance. We used the Akaike information criterion as implemented in jModelTest version 0.1 (*24*) to select the best-fit model of nucleotide substitution for the ML and Bayesian analyses. The ML tree was obtained by using PhyML version 3 (*25*), assessing branch robustness through bootstrapping (1,000 pseudo-replicates). The Bayesian summary tree was obtained by summarizing posterior tree samples generated along two 1 million generation–long Metropolis-coupled Markov chain Monte Carlo runs of 4 chains, which were performed in MrBayes version 3.1 (*26*). Convergence of the chains was assessed visually in Tracer version 1.5 (http://tree.bio.ed.ac.uk/software/tracer), and all parameters were checked to have an effective sample size of >100 in the combined run. Branch robustness was assessed through their posterior probabilities (i.e., the proportion of trees in the posterior sample in which the considered branches appeared).

### Spoligotyping, Deletion, and Principal Component Analyses

Spoligotyping was performed as described and compared with data published in SITVITWEB (*27*). Deleted regions in the chimpanzee genome with respect to H37Rv genome were inferred as regions showing a mean coverage of <50 (1% mean coverage of the genome) by using awk scripts. Principal component analysis was conducted by using BioNumerics 6.6 (www.applied-maths.com/bionumerics) with the 13,480 high-confidence variable positions used for the phylogeny. The first 3 principal components accounted for 28%, 15%, and 8% of the variability and were used to generate a 3-dimensional scatter plot (Technical Appendix Figure 4).

## Results

Necropsy of a wild chimpanzee found dead in Taï National Park, Côte d’Ivoire revealed a large, yellow-white granuloma of 5 × 6 × 3 cm in the liver and several smaller ones in the spleen parenchyma and the mesenterial lymph nodes. All other organs appeared unaffected; the lungs could not be evaluated in full because the leopard had consumed most of the tissue. 16S rDNA testing of the frozen tissue samples from the dead wild chimpanzee indicated the presence of a *Mycobacterium* sp. in various tissues. PCR–restriction fragment length polymorphism analysis confirmed MTBC in DNA preparations from spleen and mesenterial lymph nodes (*28*). MTBC was also confirmed by real-time PCR (*29*) in lung, spleen, liver, and colon abscesses.

**Figure 2:**
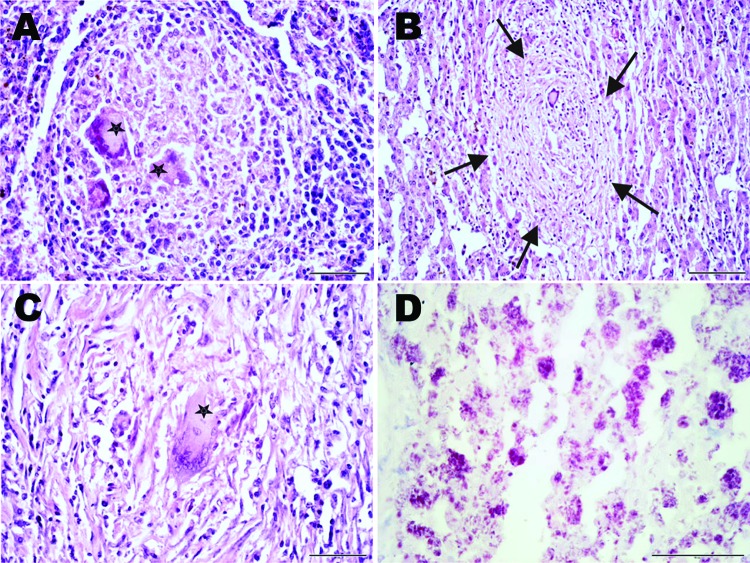
Histopathologic examination of tissue samples from adult female chimpanzee that was found dead in Taï National Park, Côte d’Ivoire, on August 5, 2009. A) Hematoxylin and eosin (H&E) stain of the spleen shows focal granulomatous inflammation with central accumulation of multinucleated Langhans giant cells (stars). B, C) H&E stain of the liver shows focal granulomatous inflammation within liver parenchyma (B, arrows) and large granulomatous alteration demarcated by fibrous connective tissue infiltrated by Langhans giant cells (C). D) Ziehl-Neelsen stain of the liver shows aggregates of acid-fast bacilli within a large granuloma. Results were consistent with *Mycobacterium tuberculosis* complex infection.

Histopathologic examination of the liver, spleen, and lymph nodes revealed a chronic granulomatous inflammation within the altered tissues (Figure 2, panels A–C). Multiple unencapsulated granulomas of varying sizes were observed in the spleen (Figure 2, panel A) and liver (Figure 2, panel B). These lesions were composed of epitheloid macrophages, few granulocytes, and multinucleated Langhans giant cells. Larger tuberculoid lesions in the liver and the lymph nodes contained a prominent central necrotic core surrounded by epitheloid cells and a few scattered Langhans giant cells. The periphery of the granulomas was demarcated by variable amounts of fibrous connective tissue and infiltrates of lymphocytes interspersed with few Langhans giant cells (Figure 2, panel C). Both intra- and extracellular acid-fast bacilli were present in the lesions (Figure 2, panel D). Taken together, these lesions were characteristic of TB and indicative of hematogenous spread and generalization of the disease.

After 23 days’ incubation, the lymph node preparations yielded typical mycobacterial colonies. The isolated MTBC strain exhibited a slow growth on Middlebrook 7H11 agar and yielded colonies after 43 days, compared with 27 days for *M. tuberculosis*. The rough surface of the colonies and the irregular spreading margins were typical features of MTBC (Technical Appendix Figure 1).

Whole-genome sequencing was conducted by using the 454 and Illumina platforms (Technical Appendix Table 1). Phylogenetic reconstruction using previously published MTBC genomes representative of the MTBC’s global diversity (*18*) confirmed that the chimpanzee strain belonged to MTBC but not to any of the known phylogenetic lineages (Figure 1). Specifically, the chimpanzee strain grouped with strains from the human-associated lineage 6, sharing 32 SNPs with this lineage, but was separate from the lineage leading to most of the animal-adapted MTBC. To further test whether the chimpanzee strain represented a new lineage rather than a variant within lineage 6, we sequenced the genome of 9 lineage 6 clinical strains from TB patients originating from different West-African countries (Technical Appendix Table 1). Our phylogenomic analysis revealed that the chimpanzee strain harboured 881 exclusive SNPs, not found anywhere else in the global MTBC phylogeny, even when including these additional lineage 6 strains. Moreover, the maximum number of SNPs between the 2 most divergent lineage 6 strains was only about half (783 SNPs) of the minimum number of differences between the chimpanzee strain and the most closely related lineage 6 strain (1,405 SNPs). When all MTBC lineages were considered, pairwise SNP distances among any 2 strains belonging to a particular lineage were always markedly lower than the minimum number of differences between the chimpanzee strain and the most closely related lineage 6 strain (Technical Appendix Figure 3).

To investigate the distinctiveness of the chimpanzee strain we isolated, we used a principal component analysis as an additional clustering method. The first 3 principal components were used to generate a 3-dimensional scatter plot (Technical Appendix Figure 4). This analysis confirmed that the chimpanzee strain did not group with lineage 6. Taken together, these results strongly suggest that the chimpanzee strain belongs to a distinct MTBC population, separate from the human-associated lineage 6.

**Figure 3:**
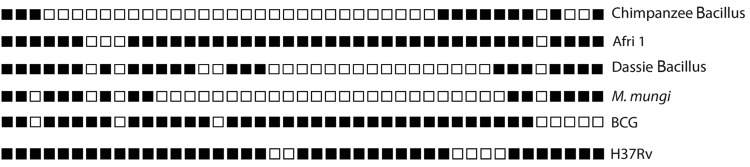
Comparison of the spoligotype of the *Mycobacterium tuberculosis* complex chimpanzee strain isolated from an adult female chimpanzee that was found dead in Taï National Park, Côte d’Ivoire, on August 5, 2009 (Chimpanzee Bacillus), with the Afri 1 spoligotype found in the most closely related human strain and the Dassie Bacillus and *M. mungi* spoligotypes described in (*12*). Spoligotypes are also shown for *M. bovis* strain BCG and human lineage 4 strain H37Rv.

To further confirm the uniqueness of the chimpanzee strain, we compared the chimpanzee spoligotype (Figure 3) with 2 large international databases that encompass >8,702 spoligotyping profiles corresponding to >58,187 MTBC isolates from global sources [*33*]; www.mbovis.org). We found that none of the spoligotyping patterns included in these databases (including 64 spoligotyping profiles from Côte d’Ivoire) matched the pattern of the chimpanzee strain (*30*) (Figure 3).

Large-sequence polymorphisms have been used as phylogenetic markers for MTBC (*4*,*7*,*8*). We found that the region of difference (RD) 9 was absent in the chimpanzee strain (*4*) (Figure 1). In addition, this strain harbored deletions in RD7, RD8, and RD10, which supports its phylogenetic relationship with lineage 6 (Technical Appendix Table 2 and Figure 2). However, the chimpanzee strain harbored only 1 of 14 lineage 6–specific deletions and did not contain the lineage 6–specific region RD900 (Technical Appendix Table 3 (*29*,*31*). Hence, this deletion-based analysis also supports a related, yet separate, phylogenetic position of the chimpanzee strain relative to lineage 6.

To investigate the possible presence of MTBC in other chimpanzees, 115 tissue samples from 28 chimpanzees that died in the same area (many from the same community) within the previous 10 years were tested by real-time PCR (14). However, no test results were positive. Consistent with the molecular analyses, necropsies and pathological evaluation of these animals revealed no signs suggestive of TB.

## Discussion

Chimpanzees are known to be susceptible to TB, and MTBC strains have been reported previously in captive chimpanzees (*32*,*33*). These 2 studies concluded that the infecting strains belonged to *M. africanum* and *M. tuberculosis*, respectively. Direct comparison of those isolates was not possible because of the limitations of typing techniques at that time. However, close contact with humans (i.e., the persons caring for these animals) suggests those captive chimpanzees were infected with human strains, as reported for other captive nonhuman primates.

By contrast, several lines of evidence support the view that the chimpanzee strain we report was not acquired from humans. First, its position on the MTBC phylogeny strongly suggests it belongs to a novel lineage. This notion is sustained by the fact that the minimum genetic distance between the chimpanzee strain and any of the nearest human strains (i.e., lineage 6) was larger than the corresponding distance between any 2 strains from the same lineage. Second, our PCA analysis showed that the chimpanzee strains did not group with lineage 6. Third, spoligotyping revealed a novel pattern among 58,187 clinical isolates from 102 countries, including Côte d’Ivoire. Fourth, genome deletion analyses corroborated the distinct phylogenetic position of the chimpanzee strain compared with known MTBC lineages. Fifth, researchers and their assistants who are in proximity of the chimpanzees at Taï National Forest are regularly screened for TB, but none has ever had a positive test result.

Two other animal-associated members of MTBC are known to cluster with lineage 6 rather than with the classical animal-adapted lineages: *M. mungi* and the Dassie Bacillus, which infect African mongooses and hyraxes, respectively (*11*,*12*). Whole genome analyses are not available for these organisms, but genomic deletion data have been reported (*34*). The chimpanzee strain we isolated did not harbor any of the specific deletions found in *M. mungi* or Dassie Bacillus (Technical Appendix Table 2) (*34*). Moreover, spoligotyping confirmed that the chimpanzee strain was distinct from *M. mungii* and Dassie Bacillus and from any other MTBC strain genotyped to date (*30*). However, 1 of the 32 SNPs shared between the chimpanzee genome and the lineage 6 strains also occurred in *M. mungii* or Dassie Bacillus. On the basis of this 1 SNP in Rv1510, which has been reported before (*5*), one could hypothesize that the chimpanzee strain and *M. mungii* and Dassie Bacillus might be related. However, genome-wide data will be necessary to define the exact phylogenetic position of *M. mungii* and Dassie Bacillus, and their relationship with the chimpanzee strain, in the global MTBC tree.

Even though chimpanzees maintain close social contacts with other members of their group, extensive necropsies and molecular screening of 28 chimpanzees from the same region yielded no additional case of TB infection, which suggests TB is rare in this chimpanzee population. This low prevalence could have several explanations. While we can likely disregard a human origin of the chimpanzee strain described here, we cannot exclude the possibility that this strain was acquired from another unidentified animal host, including other primates; chimpanzees are known to hunt other animals, including monkeys and small antelopes. The chimpanzee strain we isolated shared >1 SNP with *M. mungi* and the Dassie Bacillus, which are pathogens of 2 other small African mammals. On a more speculative note, and if it is assumed that the MTBC strain described is indeed chimpanzee-specific, this MTBC variant might be relatively attenuated and only marginally affect a chimpanzee’s health and longevity. This would enable sustained transmission and persistence of the pathogen in small host populations (*35*).

Although more work is needed to establish the prevalence, diversity, and clinical outcome of MTBC infection in wild chimpanzees and other great apes, from a conservation point of view, MTBC may join Ebola virus, *Bacillus cereus* biovar *anthracis*, and simian immunodeficiency viruses as a microorganism capable of threatening great apes in the wild (*36*). Our results suggest the effect of this MTBC strain on chimpanzee populations might be limited, but small outbreaks or single deaths can have a strong influence on the viability of isolated populations, particularly in great apes, which exhibit a slow reproductive rate and a high juvenile mortality rate (*37*).

Our study also sheds new light on the overall diversity of MTBC with implications for understanding the evolution of this pathogen. Together with other recent reports (*11*,*12*), our work suggests wider MTBC diversity, particularly among African mammals. Moreover, these data indicate that the theory that MTBC originally evolved as a human pathogen and jumped into animals is overly simplistic and may apply mainly to domestic animals. These data indicate 1 possible model could be that the common ancestor of MTBC was a generalist capable of infecting many mammals, including humans. From here, only few descendants spread around the world through human and animal migrations, creating the human-dominated phylogenetic picture we see today. We may well expect to find a much higher diversity of this MTBC, extending well outside the human-associated MTBC strains that infect various species, represented mainly through wildlife, including our closest relatives, the great apes. *M. canettii* and the other smooth TB bacilli show a high genetic diversity and are largely limited to the Horn of Africa, although whether these bacilli should be formally considered part of MTBC is controversial (*3*). Hence, they have been proposed to be part of the mycobacterial population that gave rise to the classical members of MTBC. Together with the apparent lack of human-to-human transmission of *M. canettii* (*38*), this suggestion would be consistent with a wider host range and/or environmental reservoir for the original ancestor of MTBC.

In conclusion, we report a microbiologically confirmed case of TB in a wild chimpanzee. Our molecular data show that the chimpanzee strain described here belongs to a novel lineage, more closely related to human-associated lineage 6 than to the other classical animal MTBC and possibly related to *M. mungii* and the Dassie Bacillus. This strain could represent a chimpanzee-specific pathogen or an MTBC variant acquired from another source. Because of our limited understanding of the ecology of this microbe, we propose at this stage to name it “Chimpanzee Bacillus” rather than to develop a dedicated species or subspecies name. Further studies are warranted, not only to better understand the natural history of TB in great apes and the biology of the Chimpanzee Bacillus, but also to estimate a possible risk for transmission of new types of MTBC to humans (e.g., through hunting and consumption of bushmeat). Moreover, further characterization of MTBC diversity will be crucial for understanding the origins of TB and the potential for the emergence of new strains through proximity between humans and wildlife.

Technical Appendix Sequences of *Mycobacterium tuberculosis* complex isolates from previous studies compared with isolates from this study; genomic deletions respective to the H37Rv genome in the Chimpanzee Bacillus genome; coverage of the Chimpanzee Bacillus sequence reads in *M. africanum*–specific deletions; broth culture of *M. tuberculosis* complex isolate; maximum-likelihood phylogeny of *M. tuberculosis* complex isolate; observed pairwise distance between strains within lineages of *M. tuberculosis*; and PCA scatter plot of *M. tuberculosis* complex and related strains.
