# Supplementary material for: Novel Mycobacterium tuberculosis Complex Isolate from a Wild Chimpanzee
Source: Emerg Infect Dis. 2013 Jun;19(6):969–76. doi: 10.3201/eid1906.121012 (PMC3713819; doi:10.3201/eid1906.121012)
Supplement: Technical Appendix — Sequences of Mycobacterium tuberculosis complex isolates from previous studies compared with isolates from this study; genomic deletions respective to the H37Rv genome in the Chimpanzee Bacillus genome; coverage of the Chimpanzee Bacillus sequence reads in M. africanum–specific deletions; broth culture of M. tuberculosis complex isolate; maximum-likelihood phylogeny of M. tuberculosis complex isolate; observed pairwise distance between strains within lineages of M. tuberculosis; and PCA scatter plot of M. tuberculosis complex and related strains. [file 12-1012-Techapp-s1.pdf]

Article DOI: http://dx.doi.org/10.3201/eid1906.121012

Novel *Mycobacterium tuberculosis* Complex Isolate from a Wild Chimpanzee

Technical Appendix

Technical Appendix Table 1. Sequences of *Mycobacterium tuberculosis* complex isolates from previous studies compared with isolates from this study

Isolate identification	Origin study	Lineage	GenBank accession no.
T92	(*1*)	L1	SRX003589
T17	(*1*)	L1	SRX005394
95_0545	(*1*)	L1	SRX007721
K21	(*1*)	L1	SRX002001
K67	(*1*)	L1	SRX002004
K93	(*1*)	L1	SRX002005
T67	(*1*)	L2	SRX007715
T85	(*1*)	L2	SRX003590
00_1695	(*1*)	L2	SRX007716
98_1833	(*1*)	L2	SRX007718
M4100A	(*1*)	L2	SRX007719
91_0079	(*1*)	L3	SRX007720
K49	(*1*)	L3	SRX002002
GM_1503	(*1*)	L4	SRX012272
4783_04	(*1*)	L4	SRX007723
K37	(*1*)	L4	SRX002003
544404	(*1*)	L5	SRX007725
11821_03	(*1*)	L5	SRX007724
4141_04	(*1*)	L6	SRX007726
GM_0981	(*1*)	L6	SRX007722
M. canettii	(*1*)	Outgroup	SRX002429
M. tuberculosis complex/*M. orygis**	SRA EBI	Animal	ERR015582
M.bovis (ravenel)	SRA EBI	Animal	SRR022532
541504	This study	L6	ERR031459
N0092	This study	L6	ERR031480
N0115	This study	L6	ERR031484
N0091	This study	L6	ERR031479
533604	This study	L6	ERS153831
823602	This study	L6	ERS153832
N0060	This study	L6	ERS125978
N0089	This study	L6	ERS125979
N0090	This study	L6	ERS125980
H37Rv	This study	L4	ERS153830
Chimpanzee bacillus	This study		Pending
Chimpanzee bacillus	This study		Pending

*Isolate from an antelope, recently *M. orygis* proposed for bacilli isolated from members of the Bovidae family, i.e., oryxes, gazelles, deer, antelope, and waterbucks (2). SRA EBI, European Bioinformatics Institute (www.ebi.ac.uk).

Technical Appendix Table 2. Genomic deletions respect to H37Rv genome inferred by more than 100-fold decreased coverage in the chimpanzee bacillus genome

Genome positions	Mean coverage in genome	Homologous genomic feature in H37Rv genome[a]	Genomic feature	Overlapping deletion already described
265477..265506	0	*IG223_Rv0221-Rv0222		RD10†
265507..266295	0	Rv0222	enoyl-CoA hydratase	
266296..266300	0	*IG224_Rv0222-Rv0223c		
889072..889398	1.6875	Rv0795	transposase IS6110 phage	
889395..890333	0	Rv0796	transposase IS6110 phage	
1541994..1542878	0	Rv1369c	transposase IS6110 phage	
1542879..1542928	0	*IG1392_Rv1369c-Rv1370c		
1542929..1543255	1.96429	Rv1370c	transposase IS6110 phage	
1606147..1606385	0	*IG1452_Rv1429-Rv1430		
1718726..1719970	1.4898	Rv1524	glycosyltransferase	
1719971..1720016	0	*IG1552_Rv1524-Rv1525		
1720017..1720802	0	Rv1525	*wbbL2*	
1720780..1722060	0	Rv1526c	glycosyltransferase	
1722061..1722082	0	*IG1554_Rv1526c-Rv1527c		
1779299..1779313	0	*IG1600_Rv1572c-Rv1573		RD3[†]
1779314..1779724	1	Rv1573	phiRV1 phage	
1779725..1779929	0	*IG1601_Rv1573-Rv1574		
1779930..1780241	0	Rv1574	phiRV1 phage	
1780199..1780699	0	Rv1575	phiRV1 phage	
1780643..1782064	0	Rv1576c	phiRV1 phage	
1782065..1782071	0	*IG1604_Rv1576c-Rv1577c		
1782072..1782584	1	Rv1577c	phiRV1 phage	
1782585..1782757	1	*IG1605_Rv1577c-Rv1578c		
1782758..1783228	1	Rv1578c	phiRV1 phage	
1783229..1783308	0	*IG1606_Rv1578c-Rv1579c		
1783309..1783623	0	Rv1579c	phiRV1 phage	
1783620..1783892	0	Rv1580c	phiRV1 phage	
1783893..1783905	0	*IG1608_Rv1580c-Rv1581c		
1783906..1784301	0	Rv1581c	phiRV1 phage	
1784302..1784496	0	*IG1609_Rv1581c-Rv1582c		
1784497..1785912	0	Rv1582c	phiRV1 phage	
1785912..1786310	0	Rv1583c	phiRV1 phage	
1786307..1786528	0	Rv1584c	phiRV1 phage	
1786529..1786583	0	*IG1612_Rv1584c-Rv1585c		
1786584..1787099	0	Rv1585c	phiRV1 phage	
1787096..1788505	0	Rv1586c	phiRV1 phage	
1987745..1988629	0	Rv1756c	putative transposase	
1988630..1988679	0	*IG1786_Rv1756c-Rv1757c		
1988680..1989006	1.8169	Rv1757c	putative transposase	
1989007..1989041	1	*IG1787_Rv1757c-Rv1758		
1996479..1996528	0	*IG1793_Rv1763-Rv1764		
1996529..1997413	0	Rv1764	putative transposase	
1997414..1997417	0	*IG1794_Rv1764-Rv1765c		
2208498..2208506	0	*IG1995_Rv1964-Rv1965		RD7[†]
2208507..2209322	0	Rv1965	*yrbE3b*	
2209323..2209326	0	*IG1996_Rv1965-Rv1966		
2209327..2210604	0	Rv1966	*mce3A*	
2210601..2211629	0	Rv1967	*mce3b*	
2211626..2212858	0	Rv1968	*mce3C*	
2212855..2214126	0	Rv1969	*mce3D*	
2214123..2215256	0	Rv1970	*lprM*	
2215257..2216570	0	Rv1971	*mce3F*	
2216571..2216591	0	*IG2002_Rv1971-Rv1972		
2216592..2217167	0	Rv1972	*mce* associated mem†rane protein	
2217164..2217646	0	Rv1973	*mce* associated mem†rane protein	
2217647..2217658	0	*IG2004_Rv1973-Rv1974		
2217659..2218036	0	Rv1974	hypothetical protein	
2218037..2218051	0	*IG2005_Rv1974-Rv1975		
2218052..2218717	0	Rv1975	hypothetical protein	
2218718..2218843	0	*IG2006_Rv1975-Rv1976c		
2218844..2219251	0	Rv1976c	hypothetical protein	
2219252..2219753	0	*IG2007_Rv1976c-Rv1977		

Genome positions	Mean coverage in genome	Homologous genomic feature in H37Rv genome[a]	Genomic feature	Overlapping deletion already described
2330147..2330213	1	*IG2104_Rv2072c-Rv2073c		RD9†
2330214..2330963	0	Rv2073c	shortchain dehydrogenase	
2330964..2330992	0	*IG2105_Rv2073c-Rv2074		
2330993..2331406	0	Rv2074	hypothetical protein	
2331407..2331415	0	*IG2106_Rv2074-Rv2075c		
2365465..2365791	0	Rv2105	transposase phage	
2365788..2366726	1	Rv2106	transposase phage	
2430159..2431199	1	Rv2167c	transposase phage	
2431094..2431420	0	Rv2168c	transposase phage	
2550030..2550064	0	*IG2310_Rv2277c-Rv2278		
2550065..2550391	0	Rv2278	transposase phage	
2550388..2551326	0	Rv2279	transposase phage	
2635593..2635627	0	*IG2392_Rv2353c-Rv2354		
2635628..2635954	1	Rv2354	transposase phage	
2635951..2636889	1	Rv2355	transposase phage	
2784657..2785697	0	Rv2479c	transposase phage	
2785592..2785918	1.23077	Rv2480c	transposase phage	
2972160..2972486	1	Rv2648	transposase IS6110 phage	
2972435..2973421	0	Rv2649	transposase IS6110 phage	
3120566..3121504	0	Rv2814c	transposase phage	
3121501..3121827	0	Rv2815c	transposase phage	
3551281..3551607	0	Rv3184	transposase phage	
3551604..3552542	0	Rv3185	transposase phage	
3552543..3552763	70.2409	*IG3235_Rv3185-Rv3186		
3552764..3553090	0	Rv3186	transposase phage	
3553087..3554025	0	Rv3187	transposase phage	
3710756..3711694	0	Rv3326	transposase phage	
3795100..3795984	1	Rv3380c	transposase phage	
3795985..3796034	0	*IG3438_Rv3380c-Rv3381c		
3796035..3796361	23.0909	Rv3381c	transposase phage	
3890830..3891156	1	Rv3474	transposase IS6110 phage	
3891051..3892091	0	Rv3475	transposase IS6110 phage	
4057733..4058701	0	Rv3617	EphA	RD8†
4058698..4059885	0	Rv3618	monooxygenase	
4059886..4059983	3	*IG3678_Rv3618-Rv3619c		
4060592..4060647	0	*IG3680_Rv3620c-Rv3621c		
4060648..4061889	0	Rv3621c	PPE65	
4061890..4061898	0	*IG3681_Rv3621c-Rv3622c		
4061899..4062198	0	Rv3622c	PE32	
4062199..4062526	0	*IG3682_Rv3622c-Rv3623		
4343314..4345035	1.31683	Rv3868	hypothetical protein	
4345036..4345038	0	*IG3933_Rv3868-Rv3869		

*IG account for intergenic regions.
†Genomic deletion identified in Gagneux et al. (*3*).

Technical Appendix Table 3. Coverage of the chimpanzee bacillus sequence reads in *M. africanum* specific deletions described in Mostowy et al. (*4*)

Deletion	Mean coverage
RD720	4546.4117
RD721	4728.18
RD701	1440.11
RD702	3991
RD722	4310.79
RD711	4695.3543
RD742	3709.58
RD713	3252.15
RD743	4693.5488
RD724	4145.295
RD728	4749.474
RD715	0.6
RD727	4559.3533
RD735	5045.54

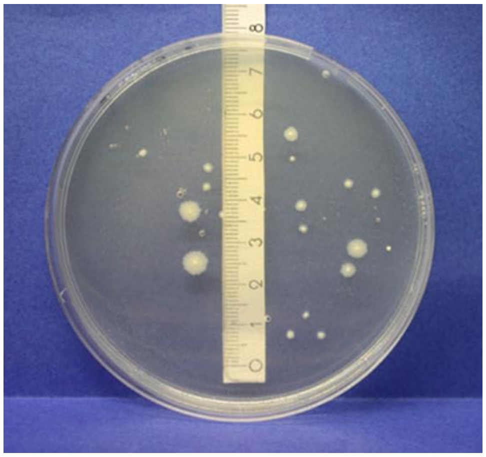

Technical Appendix Figure 1. Broth culture of *Mycobacterium tuberculosis* complex isolate, diluted, plated onto Middlebrook 7H11 agar, and photographed after 76 days.

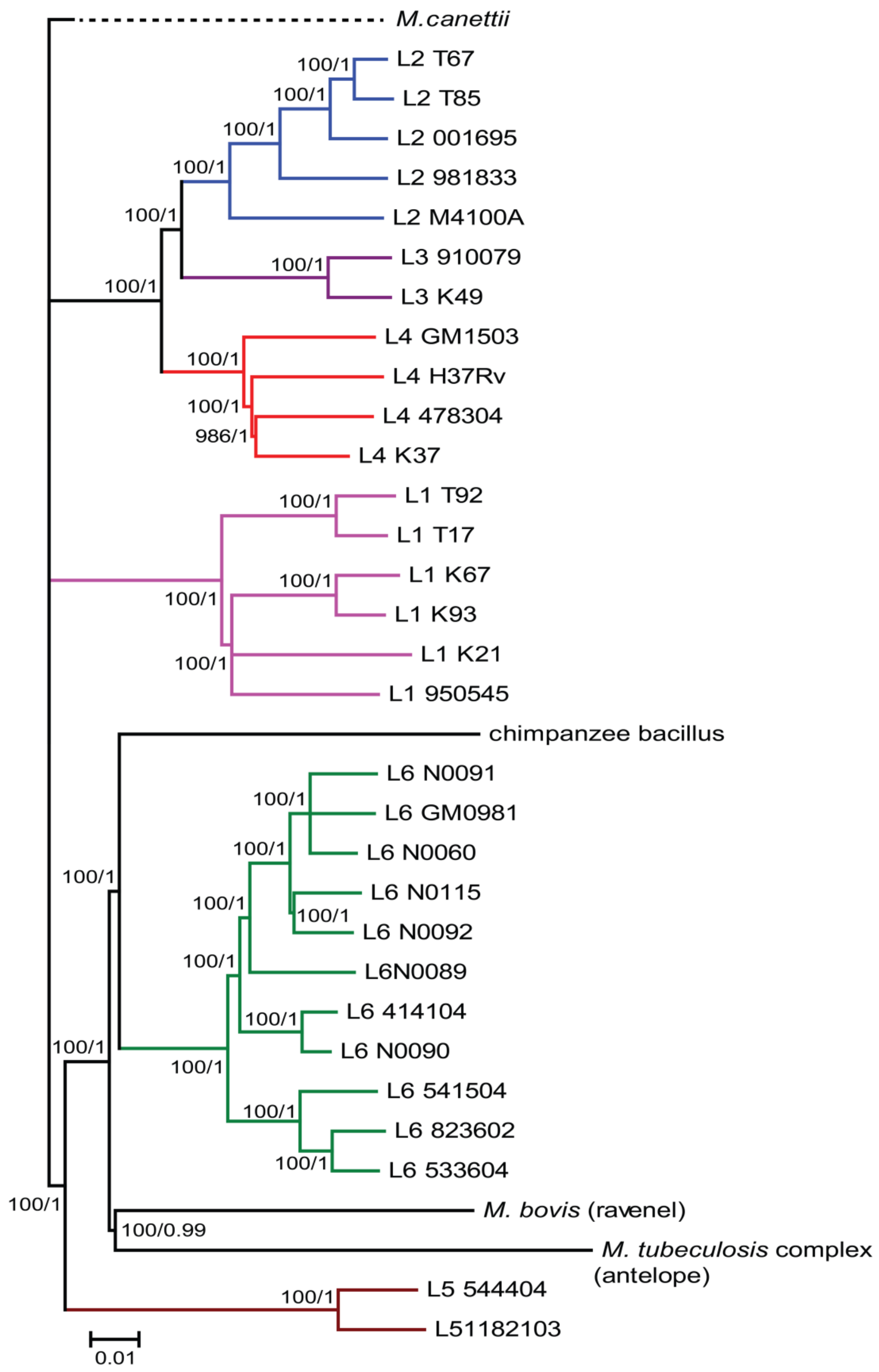

Technical Appendix Figure 2. Maximum-likelihood phylogeny of *Mycobacterium tuberculosis* complex isolate, obtained by using PhymL (5) and 1,000 bootstrap pseudoreplicates for clade support. Posterior probabilities for each node by Bayesian-inference tree (6) are place next to the bootstrap values.

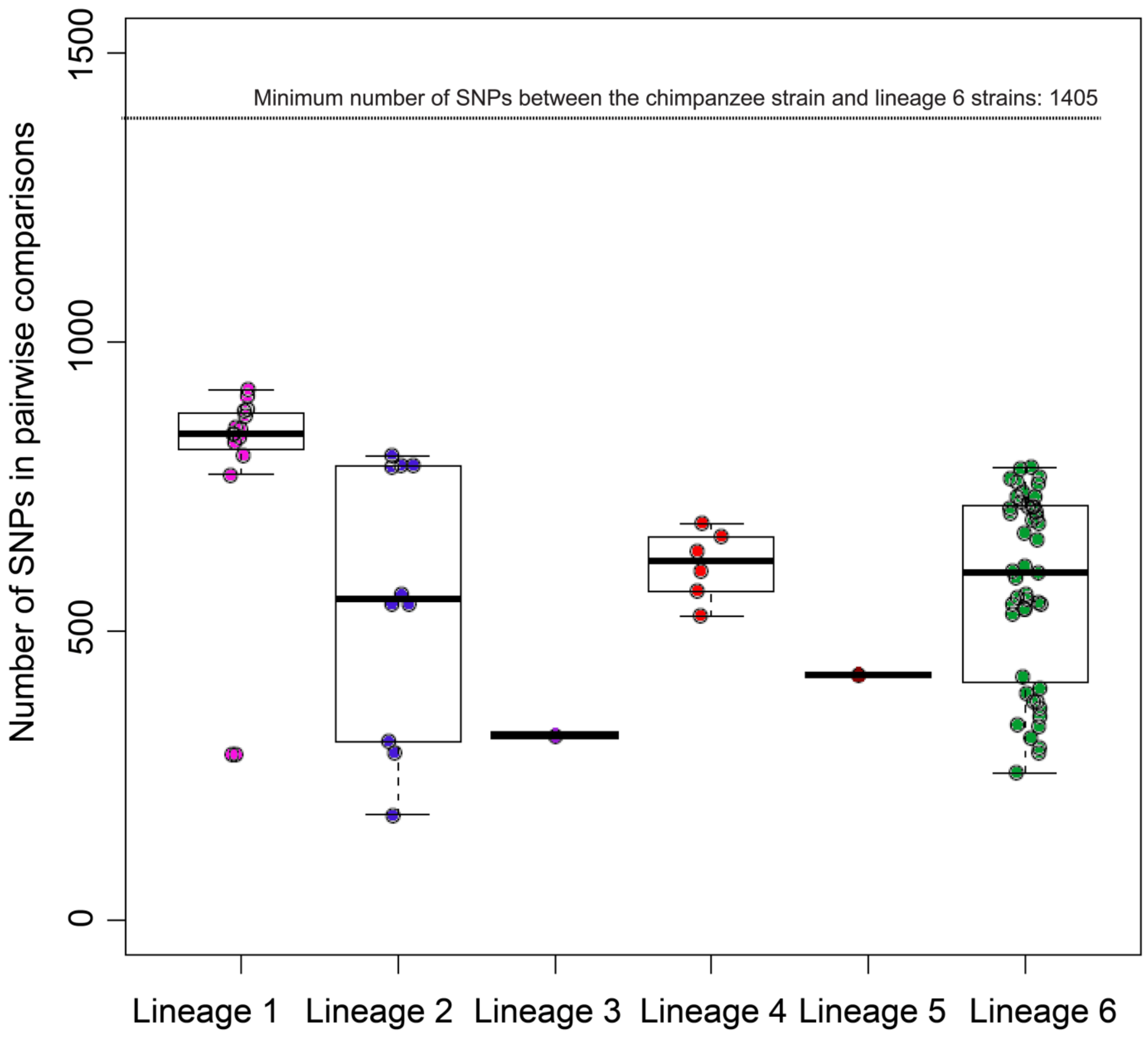

Technical Appendix Figure 3. Observed pairwise distance between strains within lineages of *Mycobacterium tuberculosis*. For box plots: median (horizontal line), interquartile range (box), minimum and maximum values (whiskers), and outliers (circles). The minimum number of single-nucleotide polymorphisms (SNPs) between the chimpanzee strain and the closest group (Lineage 6/*M. africanum* WA2) is shown as a dotted line; note that this distance is larger than the maximum pairwise distance within any of the lineages.

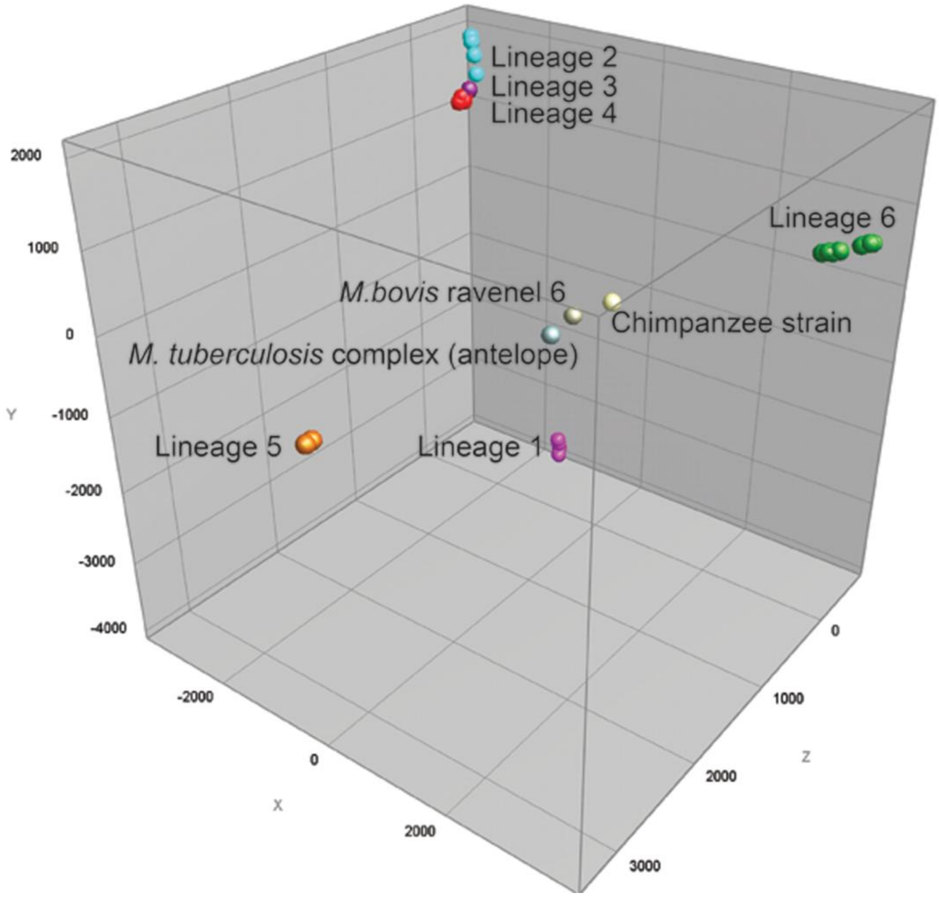

Technical Appendix Figure 4. PCA scatter plot of *Mycobacterium tuberculosis* complex and related strains, based on 13,480 variable nucleotide positions. Only the first three principal components from the PCA were used. Strains are grouping according to lineages, and the chimpanzee strain is not grouping with Lineage 6.

References

1. Li H, Durbin R. Fast and accurate short read alignment with Burrows Wheeler transform. Bioinformatics. 2009;25:1754–60. PubMed http://dx.doi.org/10.1093/bioinformatics/btp324

2. van Ingen J, Rahim Z, Mulder A, Boeree MJ, Simeone R, Brosch R, et al. Characterization of *Mycobacterium orygis* as *M. tuberculosis* complex subspecies. Emerg Infect Dis. 2012;18:653–5. PubMed http://dx.doi.org/10.3201/eid1804.110888

3. Gagneux S, DeRiemer K, Van T, Kato-Maeda M, de Jong BC, Narayanan S, et al. Variable host-pathogen compatibility in *Mycobacterium tuberculosis.* Proc Natl Acad Sci U S A. 2006;103:2869–73. PubMed http://dx.doi.org/10.1073/pnas.0511240103

4. Mostowy S, Onipede A, Gagneux S, Niemann S, Kremer K, Desmond EP, et al. Genomic analysis distinguishes *Mycobacterium africanum.* J Clin Microbiol. 2004;42:3594–9. PubMed http://dx.doi.org/10.1128/JCM.42.8.3594-3599.2004

5. Guindon S, Lethiec F, Duroux P, Gascuel O. PHYML Online—a web server for fast maximum likelihood-based phylogenetic inference. Nucleic Acids Res. 2005;33:W557–9. PubMed http://dx.doi.org/10.1093/nar/gki352

6. Ronquist F, Huelsenbeck JP. MrBayes 3: Bayesian phylogenetic inference under mixed models. Bioinformatics. 2003;19:1572–4. PubMed http://dx.doi.org/10.1093/bioinformatics/btg180
